# Gene Therapy to Treat Osteopenia Associated With Chronic Ethanol Consumption and Aldehyde Dehydrogenase 2 Deficiency

**DOI:** 10.1002/jbm4.10723

**Published:** 2023-02-16

**Authors:** Anna E Camilleri, Michelle Cung, Fiona M Hart, Odelya E Pagovich, Ronald G Crystal, Matthew B Greenblatt, Katie M Stiles

**Affiliations:** ^1^ Department of Genetic Medicine Weill Cornell Medical College New York NY USA; ^2^ Pathology and Laboratory Medicine Weill Cornell Medical College New York NY USA; ^3^ Research Division Hospital for Special Surgery New York NY USA

**Keywords:** GENETIC ANIMAL MODELS, OSTEOPOROSIS, BONE QCT/MICROCT

## Abstract

Aldehyde dehydrogenase 2 (ALDH2) deficiency affects 35% to 45% of East Asians and 8% of the world population. ALDH2 is the second enzyme in the ethanol metabolism pathway. The common genetic variant ALDH2*2 allele has a glutamic acid‐to‐lysine substitution at position 487 (E487K) that reduces the enzyme activity, resulting in an accumulation of acetaldehyde after ethanol consumption. The ALDH2*2 allele is associated with increased risk of osteoporosis and hip fracture. Our prior study showed that administration of an adeno‐associated virus (AAV) serotype rh.10 gene transfer vector expressing the human *ALDH2* cDNA (AAVrh.10hALDH2) before initiation of ethanol consumption prevented bone loss in ALDH2‐deficient homozygous knockin mice carrying the E487K mutation (*Aldh2* ^E487K+/+^). We hypothesized that AAVrh.10hALDH2 administration after establishment of osteopenia would be able to reverse bone loss due to ALDH2 deficiency and chronic ethanol consumption. To test this hypothesis, male and female *Aldh2* ^E487K+/+^ mice (*n* = 6) were given ethanol in the drinking water for 6 weeks to establish osteopenia and then administered AAVrh.10hALDH2 (10^11^ genome copies). Mice were evaluated for an additional 12 weeks. AAVrh.10hALDH2 administration after osteopenia was established corrected weight loss and locomotion phenotypes and, importantly, increased midshaft femur cortical bone thickness, the most important component of bone in the resistance to fractures, and showed a trend toward increased trabecular bone volume. AAVrh.10hALDH2 is a promising therapeutic for osteoporosis in ALDH2‐deficient individuals. © 2023 The Authors. *JBMR Plus* published by Wiley Periodicals LLC on behalf of American Society for Bone and Mineral Research.

## Introduction

Aldehyde dehydrogenase 2 (ALDH2) deficiency is one of the most common hereditary disorders affecting 560 million people worldwide.^(^ ^1^, ^2^ ^)^ The prevalence is highest in individuals of East Asian descent (35% to 45%).^(^ ^2^, ^3^ ^)^ ALDH2 is localized to the mitochondria and is most abundantly expressed in the liver.^(^ ^4^, ^5^ ^)^ ALDH2 metabolizes aldehydes and is the second enzyme in the ethanol metabolism pathway converting acetaldehyde to acetate.^(^ ^6^, ^7^ ^)^ With ethanol ingestion, there is substantial serum acetaldehyde accumulation in patients bearing mutations that reduce the oxidizing ability of ALDH2.^(^ ^3^, ^8^, ^9^ ^)^ ALDH2 is a tetramer and the mutant protein functions as a dominant negative; heterozygotes have <50% enzymatic activity and homozygotes <4%.^(^ ^10^, ^11^ ^)^ A glutamic acid‐to‐lysine substitution at position 487 (E487K; ALDH2*2 allele) is the most common mutation and is responsible for the “Asian flush syndrome.”^(^ ^3^, ^11^, ^12^, ^13^ ^)^ ALDH2 E487K mutant protein has reduced ability to oxidize acetaldehyde and has an increased protein turnover rate even though the expression at the mRNA level is the same as the wild‐type ALDH2.^(^ ^9^, ^14^, ^15^ ^)^ With chronic ethanol consumption, the ALDH2 E487K is also linked to many serious chronic disorders, including a highly elevated risk of cancers of the upper aerodigestive tract^(^ ^16^, ^17^, ^18^, ^19^, ^20^, ^21^, ^22^ ^)^ and osteoporosis and hip fracture.^(^ ^23^, ^24^ ^)^

Consistent with human ALDH2 deficiency, chronic ethanol consumption in ALDH2‐deficient *Aldh2* knockout mice (*Aldh2* ^−/−^), transgenic mice overexpressing the ALDH2*2 allele, or *Aldh2* ^E487K+/+^ knockin mice expressing a murine ALDH2 bearing the E487K mutation from human ALDH2*2 all result in decreased bone mass compared with wild‐type animals.^(^ ^20^, ^25^, ^26^ ^)^ Our previous study demonstrated that prophylactic treatment of *Aldh2* ^−/−^ and *Aldh2* ^E487K+/+^ ALDH2‐deficient mice with an adeno‐associated virus serotype rh.10 coding for wild‐type human ALDH2 (AAVrh.10hALDH2) before initiation of ethanol consumption could prevent chronic ethanol‐induced phenotypes, including weight loss, progressively decreased locomotion and coordination, and, importantly, bone loss in the trabecula and midshaft cortex of the femur.^(^ ^26^ ^)^ In the present study, we hypothesized that AAVrh.10hALDH2 treatment after osteopenia has been established would be able to reverse bone loss associated with ALDH2 deficiency and chronic ethanol consumption.

## Materials and Methods

### Mouse model of ALDH2 deficiency

*Aldh2* E487K knockin homozygous mice (*Aldh2* ^E487K+/+^), a knockin mouse model of ALDH2 deficiency bearing an inactivating point mutation from the human ALDH2*2 allele in the mouse *Aldh2* gene, were obtained from the Department of Chemical and Systems Biology, Stanford University School of Medicine (Stanford, CA, USA).^(^ ^27^ ^)^ All mice were housed in microisolator cages (2 to 4 mice/cage) and all food and water were autoclaved. Homozygous mice were bred as pairs (one female with one male) or trios (two females with one male) and genotyping was confirmed in pups at 3 weeks of age by PCR (Transnetyx, Cordova, TN, USA) using primers: forward–5′‐GGAGCTGGGCGAGTATGG, reverse–5′‐GAGTCTGAAGGCTGTGTACGTA.^(^ ^10^, ^27^ ^)^ All experiments conformed to the relevant regulatory standards and were approved by the Institutional Animal Care and Use Committee of Weill Cornell Medical College.

### AAV vectors

The AAVrh.10hALDH2 vector is composed of the nonhuman primate‐derived rh.10 capsid and an expression cassette including the 5′ and 3′ AAV2 inverted terminal repeats, the cytomegalovirus (CMV) enhancer, chicken–β‐actin promoter and intron, and rabbit β globin splice acceptor (CAG promoter), the human *ALDH2* coding sequence with a hemagglutinin (HA) tag, and rabbit β‐globin polyadenylation signal.^(^ ^28^ ^)^ The HA tag was used for differentiating between human and mouse ALDH2 proteins because the mouse and human amino acid sequences are 96% homologous (NCBI Homologene: https://www.ncbi.nlm.nih.gov/homologene/55480). The vector was produced using human embryonic kidney 293T cells as described previously.^(^ ^29^ ^)^ Briefly, the pAAV‐CAG‐hALDH2‐HA expression plasmid (600 μg) and the AAVrh.10 packaging‐Ad helper hybrid plasmid pPAKMArh.10 (1200 μg) were co‐transfected into 293 T cells using PEI transfection reagent (Polysciences, Warrington, PA, USA). At 72 hours post‐transfection, cells were harvested, and lysate prepared using five freeze/thaw cycles. The cell lysate containing the virus was clarified by centrifugation at 2675 x g for 15 minutes. The AAVrh.10hALDH2 vector was purified from the crude viral lysate by iodixanol gradient and QHP anion exchange chromatography (GE Healthcare, Piscataway, NJ, USA), concentrated using a Vivaspin 100 K membrane concentrator (Cytiva, Marlborough, MA, USA) and stored in phosphate‐buffered saline, pH 7.4 at −80°C.

Vector genome titers were determined by TaqMan qPCR using a CAG specific primer–probe set (forward primer: 5′‐GTCAATGGGTGGAGTATTTACGG, reverse primer: 5′‐AGGTCATGTACTGGGCATAATGC) (Applied Biosystems, Foster City, CA, USA). The purified AAVrh.10hALDH2 vector was digested with proteinase K in the presence of 0.5% sodium dodecyl sulfate (SDS) plus 25 mM ethylenediaminetetraacetate (EDTA) at 70°C for 1 hour followed by protease inactivation at 95°C for 15 minutes. The vector was then used as a template for TaqMan analysis using a pAAV‐CAG‐hALDH2‐HA plasmid DNA standard of known copy number to generate a standard curve. The AAVrh.10control vector expresses an irrelevant transgene.^(^ ^30^ ^)^

### Overview of efficacy studies with AAVrh.10hALDH2

Male and female *Aldh2* ^E487K+/+^ mice (*n* = 6), age 10 weeks, were given water or ethanol in water *ad libitum* in the drinking water for 6 weeks (10% ethanol in water). Mice were then administered a 100 μL one‐time dose of AAVrh.10hALDH2 (10^11^ gc), AAVrh.10control (10^11^ gc), or PBS via intravenous injection. Mice were assigned to groups randomly. At this time point, the ethanol challenge was increased to 15% ethanol in water for the remaining 12 weeks. This ethanol challenge protocol was consistent with previous studies.^(^ ^26^ ^)^ Mice were evaluated for body weight and muscular coordination and strength (rotarod test) every 3 weeks. At 12 weeks post‐vector administration (18 weeks total ethanol ingestion), mice were euthanized. Liver h*ALDH2* mRNA was evaluated by TaqMan RT‐qPCR. ALDH2 enzymatic activity in liver was quantified using a commercially available assay kit (Abcam; ab115348, Cambridge, MA, USA). Osteopenia in the femur was assessed by micro‐computed tomography (μCT). Serum levels of bone marker PINP were assessed using a commercially available ELISA assay. No adverse events were noted in any experimental groups, and there were no deviations from the described protocol except that the livers from three mice in the study could not be used for mRNA and ALDH2 activity analysis because of incorrect processing (2 female PBS control, 1 female AAVrh.10control). The accurate number of samples processed for each assay is noted with the method description.

### ALDH2 expression in liver

Human *ALDH2* mRNA levels in the liver were assessed by TaqMan RT‐qPCR using FAM dye–labeled h*ALDH2*‐specific primer‐probe sets (Assay ID; Hs01007998_ml) and murine 18 S specific primer‐probe sets (Life Technologies, Waltham, MA USA) and compared with a standard curve derived from the pAAV‐CAG‐hALDH2‐HA expression plasmid. For male mice, *n* = 6 mice were processed for all groups; for female mice, AAVrh.10hALDH2 treated (*n* = 6), AAVrh.10control treated (*n* = 5), PBS treated (*n* = 4). The enzymatic activity of ALDH2 in liver was analyzed using the colorimetric mitochondrial aldehyde dehydrogenase (ALDH2) activity assay kit (Abcam; ab115348) according to the manufacturer's protocol using 200 μg of total protein from liver homogenate. For male mice, ethanol plus AAVrh.10hALDH2 (*n* = 6), ethanol plus AAVrh.10control (*n* = 6), ethanol plus PBS (*n* = 6), and water plus PBS (*n* = 5); for female mice, ethanol plus AAVrh.10hALDH2 (*n* = 6), ethanol plus AAVrh.10control (*n* = 5), ethanol plus PBS (*n* = 4), and water plus PBS (*n* = 6).

### Assessment of body weight and locomotion

To assess the effects of chronic ethanol challenge on total body mass, mice were weighed weekly. The rotarod behavior test was used to evaluate mouse strength and coordination every 3 weeks during ethanol challenge. An automated 4‐lane rotarod unit (AccuScan Instruments, Columbus, OH, USA) was used to evaluate locomotor activity. The apparatus has 7‐cm‐diameter drums with grooves to improve grip. Drums were rotated at a fixed speed of 2 rotations/minute (RPM) for the first 20 seconds, accelerated up to 30 RPM in the next 100 seconds and then up to 60 RPM in the last 60 seconds. The time and RPM when the mouse fell from or failed to walk on a drum was recorded. Failure to walk was defined as a mouse that did not fall from the drum but clung in one position and went around twice. Tests were performed twice at each time point and the average RPM was calculated. Six mice were measured for all groups.

### μCT and bone serum marker analysis

Femurs fixed in 4% paraformaldehyde and stored in 70% ethanol were scanned using a high‐resolution Scanco μCT 35 (Scanco Medical AG, Bruttisellen, Switzerland). Specimens were scanned in 70% ethanol with an isotropic voxel size of 7 μm and an intensity of 145 μA. For analysis of femoral bone mass, a region of trabecular bone 2.1 mm wide was manually contoured following an irregular shape a few pixels away from the endocortical surface, starting 280 μm from the proximal end of the distal femoral growth plate. Femoral trabecular bone was thresholded at 435.2 mg HA/ccm and femoral cortical bone was thresholded at 585.3 mg HA/ccm. A Gaussian noise filter optimized for murine bone was applied to reduce noise in the thresholded 2D image. 3D reconstructions were created by stacking the thresholded, 2D images from the contoured regions.^(^ ^31^ ^)^ Six mice were measured for all groups for μCT measurements.

N‐terminal propeptide of type I procollagen (PINP) levels were assessed in serum collected 6 weeks post‐administration of AAVrh.10hALDH2 (week 12 of ethanol challenge) using the Rat/Mouse PINP EIA ELISA (Immunodiagnostic Systems; AC33‐F1; East Bolden, UK) according to the manufacturer's instructions. Six mice were analyzed for all groups except the female ethanol plus AAVrh.10hALDH2 that had 5 because there was not sufficient serum obtained for the assay from one animal in the group.

### Statistical analysis

G*Power3.1.9.2 software (Universität Düsseldorf, Düsseldorf, Germany) was used to calculate sample size. Using a post hoc power analysis for ANOVA, the achieved power for the cortical bone thickness measurements was >0.99 with groups of *n* = 6. All data are presented as means ± standard deviation (SD); the *n* value for each group is stated in the methods section for each assay. Differences between groups were analyzed using a one‐way ANOVA with Tukey's multiple comparisons test. Any *p* values <0.05 were considered significant for all comparisons.

## Results

### AAVrh.10hALDH2‐mediated expression of h*ALDH2*

**Fig. 1:**
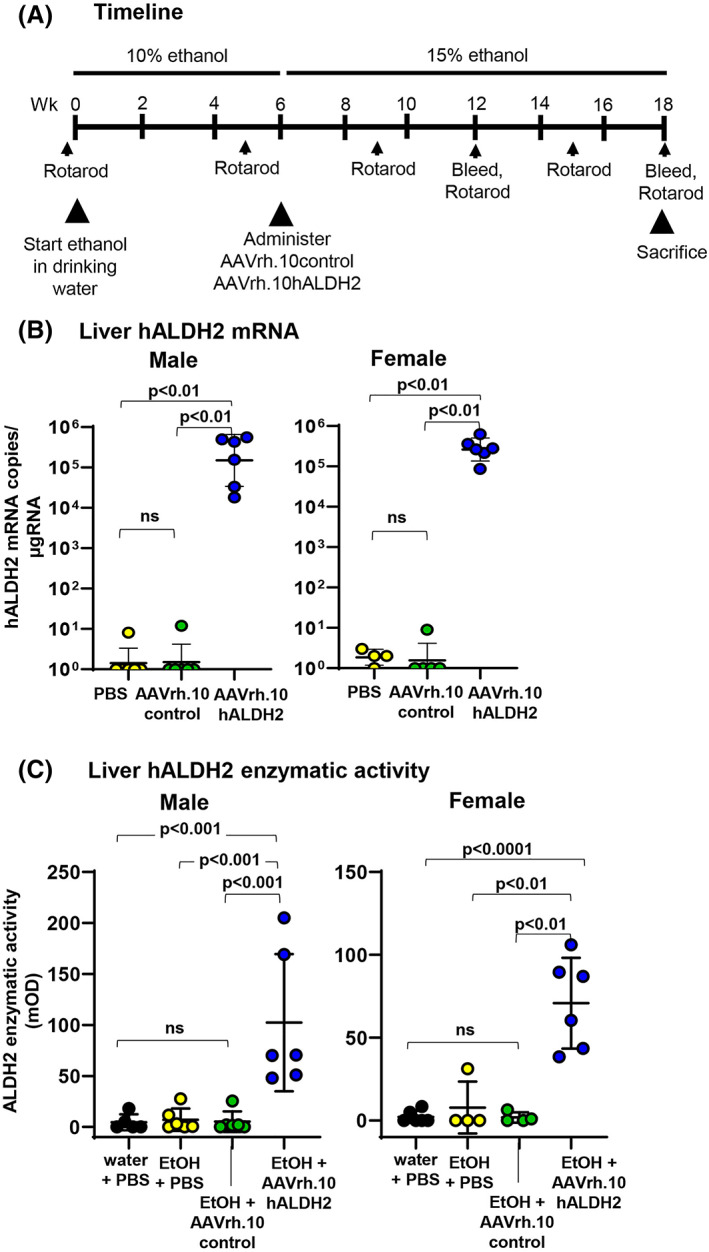
Administration of AAVrh.10hALDH2 to ALDH2‐deficient mice with osteopenia. Mice were challenged with water or ethanol for 18 weeks. Six weeks after beginning ethanol challenge, *Aldh2* ^E487K+/+^ mice were intravenously administered AAVrh.10hALDH2 (10^11^ gc), AAVrh.10control (10^11^ gc), or PBS. (*A*) Timeline of experiment. (*B*) Liver mRNA expression at 18 weeks (12 weeks post‐vector administration) assessed by TaqMan RT‐qPCR. (*C*) Liver ALDH2 enzymatic activity at week 18. Data are presented as means ± SD.

To determine if AAVrh.10hALDH2 could reverse established osteopenia in ALDH2‐deficient mice, *Aldh2* ^E487K+/+^ mice were treated with AAVrh.10hALDH2 or AAVrh.10control (10^11^ gc) or PBS by intravenous administration 6 weeks after beginning *ad libitum* ethanol in the drinking water, and then ethanol was continued for an additional 12 weeks (Fig. 1*A*). At 18 weeks, h*ALDH2* mRNA and ALDH2 enzymatic activity in liver were analyzed. AAVrh.10hALDH2‐treated *Aldh2* ^E487K+/+^ mice had significantly higher h*ALDH2* mRNA expression in the liver than AAVrh.10control‐treated mice (males *p* < 0.01, females *p* < 0.01; Fig. 1*B*) or PBS treated mice (males *p* < 0.01, females *p* < 0.01). AAVrh.10control‐treated or PBS‐treated mice had undetectable levels of ALDH2 liver enzyme activity, whereas AAVrh.10hALDH2‐treated male and female mice showed high levels of enzymatic activity (versus AAVrh.10control: males *p* < 0.001, females *p* < 0.01; versus PBS: males *p* < 0.01, females *p* < 0.01; Fig. 1*C*) comparable to the levels reported in our previous studies for male mice.^(^ ^26^, ^28^ ^)^ There was no significant difference in the levels of ALDH2 mRNA or ALDH2 activity between male and female mice.

### Body weight and locomotion assessment

**Fig. 2:**
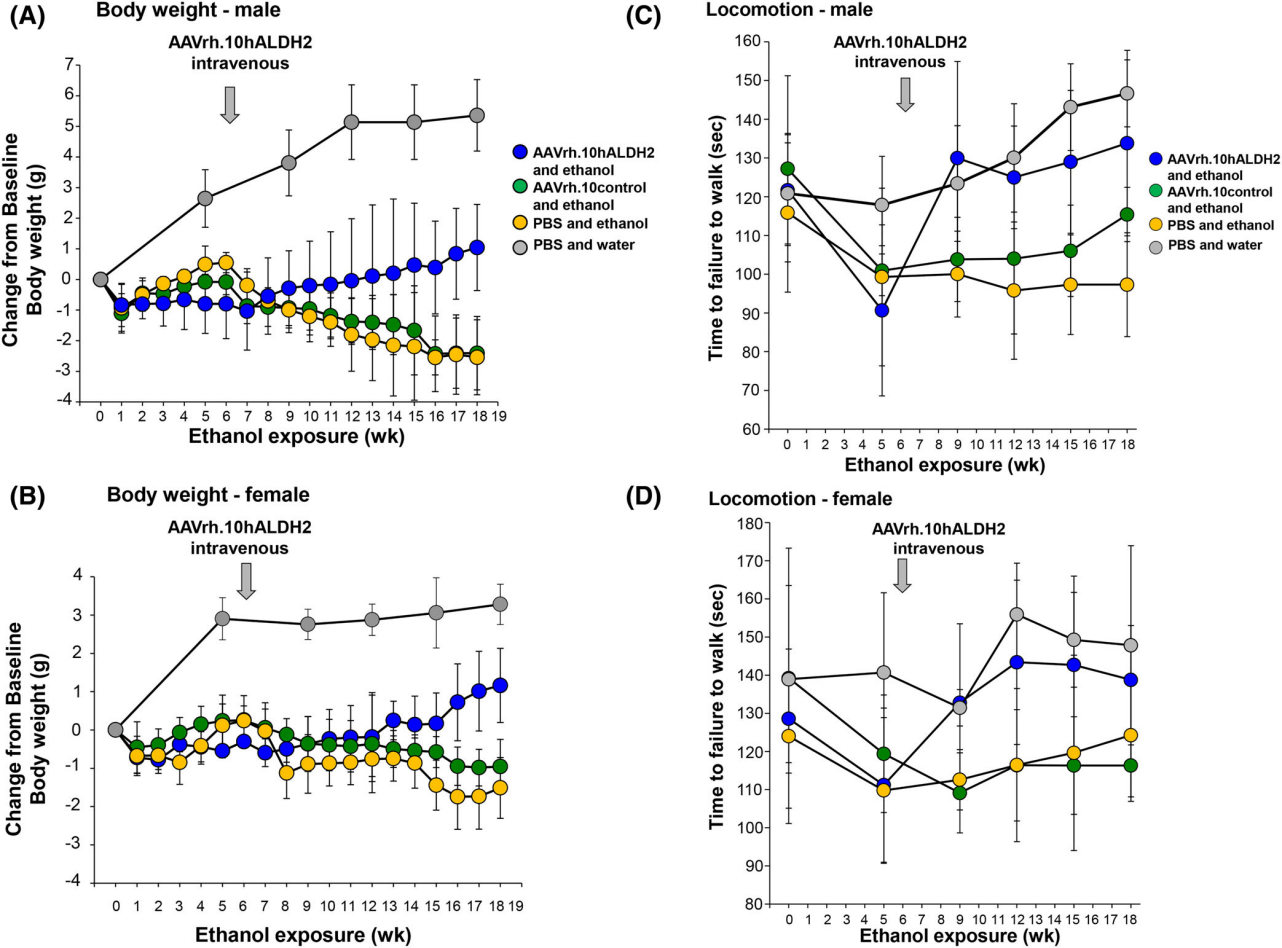
Effect of AAVrh.10hALDH2 therapy on body weight and locomotion during chronic ethanol exposure. Mice were challenged with water or ethanol for 18 weeks. Six weeks after initiation of ethanol administration, *Aldh2* ^E487K+/+^ mice were intravenously administered AAVrh.10hALDH2 (10^11^ gc), AAVrh.10control (10^11^ gc), or PBS. Tests were performed pre‐exposure and every 3 weeks during ethanol exposure. Locomotion (rotarod) assessment of maximum rotations per minute (RPM) without falling. (*A*) Change from baseline body weight—males. (*B*) Change from baseline body weight—females. (*C*) Locomotion—males. (*D*) Locomotion—females. Values are presented as means ± SD. Statistical analyses for (*A–D*) for all groups at each time point are found in Supplemental Tables S1–S4.

The body weight of the mice was evaluated every week. Body weight of AAVrh.10control‐treated and PBS male and female *Aldh2* ^E487K+/+^ mice given ethanol decreased significantly over time (male: Fig. 2*A* and Supplemental Fig. S1*A*; female: Fig. 2*B* and Supplemental Fig. S1*B*). There was no significant difference in the percent of body weight lost in male and female mice in the control groups. AAVrh.10hALDH2‐treated mice given ethanol had significantly greater weight gain and were comparable to *Aldh2* ^E487K+/+^ mice given water for increase in body weight from 6 to 18 weeks (Supplemental Fig. S1*A, B*, Supplemental Tables S1 and S2). Mice were tested on the rotarod test of locomotion before vector administration (5 weeks) and then every 3 weeks post‐administration (weeks 9, 12, 15, 18). Before vector administration, male and female *Aldh2* ^E487K+/+^ mice given ethanol had poor performance on the rotarod compared with mice given water (male: Fig. 2*C*; female: Fig. 2*D*). The rotarod performance of PBS or AAVrh.10control‐treated mice remained poor out to 18 weeks. However, the rotarod performance for *Aldh2* ^E487K+/+^ mice treated with AAVrh.10hALDH2 recovered by 9 weeks and was similar to the *Aldh2* ^E487K+/+^ mice given water through 18 weeks (Supplemental Tables S3 and S4).

### Bone treatment data in AAVrh.10hALDH2‐treated ALDH2‐deficient mice

**Fig. 3:**
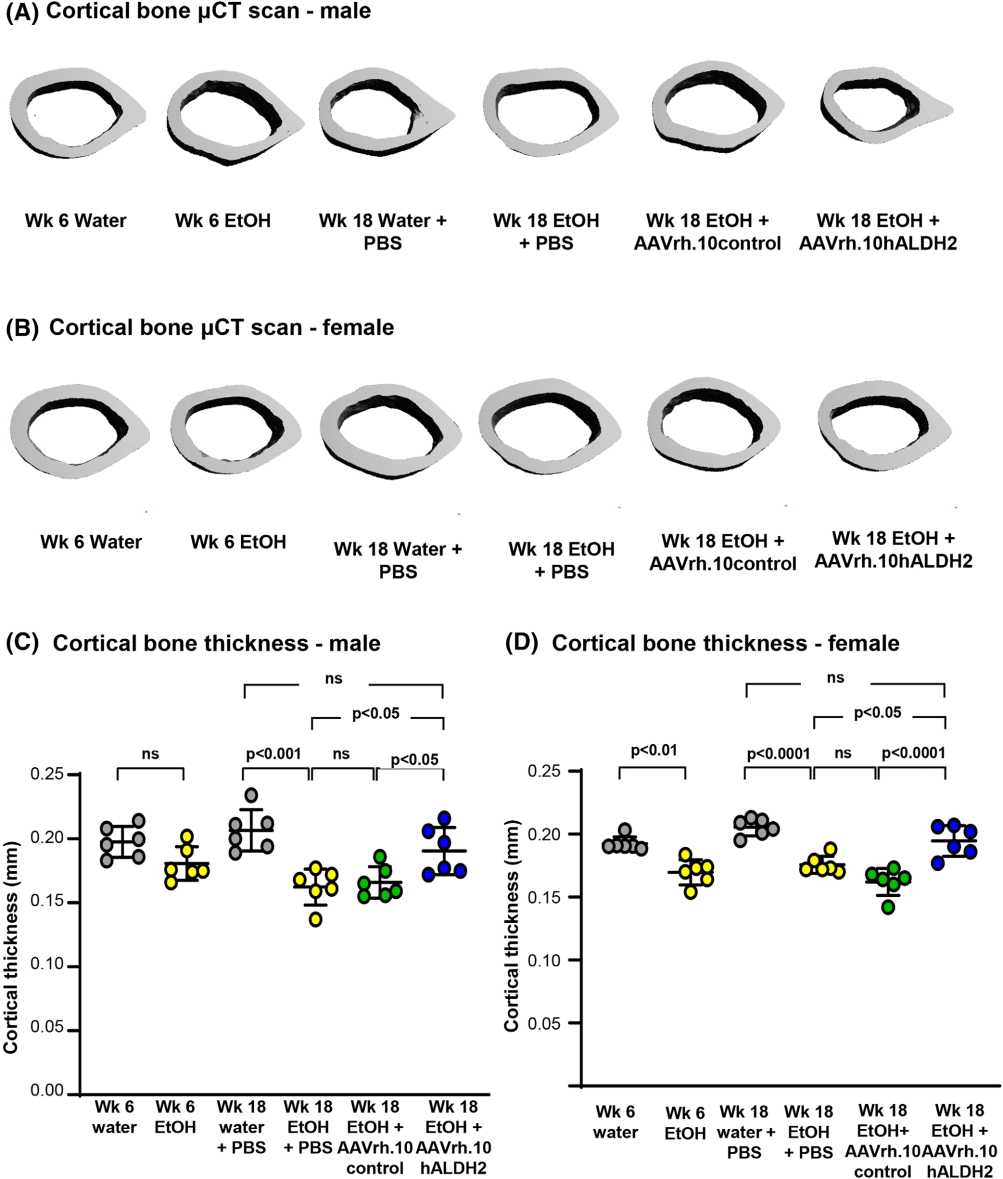
Three‐dimensional μCT reconstructions of femoral cortical bone for AAVrh.10hALDH2 therapy after chronic ethanol ingestion. *Aldh2* ^E487K+/+^ mice were challenged with water or ethanol for 18 weeks in total. Six weeks post‐initiation of ethanol administration, mice were intravenously administered AAVrh.10hALDH2 (10^11^ gc), AAVrh.10control (10^11^ gc), or PBS. Fixed femurs were analyzed by μCT. Shown are representative 3D reconstructions of midshaft cortical bone from one representative animal of each group. (*A*) Cortical bone—males. (*B*) Cortical bone—females. Quantification of cortical bone thickness (mm): (*C*) males; (*D*) females. Values are presented as means ± SD. EtOH = ethanol.

**Fig. 4:**
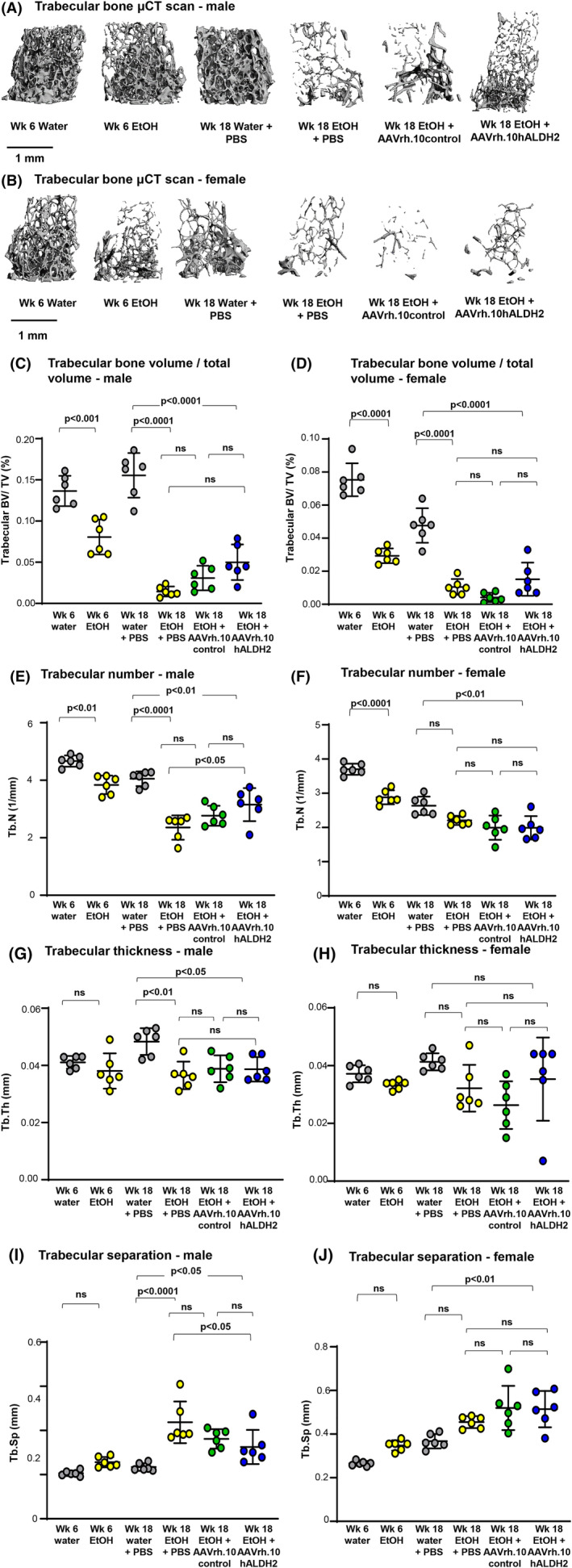
Three‐dimensional μCT reconstructions of femur trabecular bone for AAVrh.10hALDH2 therapy after chronic ethanol ingestion. *Aldh2* ^E487K+/+^ mice were challenged with water or ethanol for 18 weeks total. Six weeks post‐initiation of ethanol administration, mice were intravenously administered AAVrh.10hALDH2 (10^11^ gc), AAVrh.10control (10^11^ gc), or PBS. Fixed femurs were analyzed by μCT. Shown are representative 3D reconstructions of trabecular bone in metaphysis from one representative animal of each group. (*A*) Trabecular bone—males. (*B*) Trabecular bone—females. Quantitative μCT assessment of AAVrh.10hALDH2 therapy on bone structure of femurs: (*C*) trabecular bone volume/total volume—males; (*D*) trabecular bone volume/total volume—females. (*E*) Trabecular number—males. (*F*) Trabecular number—females. (*G*) Trabecular thickness—males. (*H*) Trabecular thickness—females. (*I*) Trabecular space—males. (*J*) Trabecular space—females. Values are presented as means ± SD. EtOH = ethanol.

AAVrh.10hALDH2 prophylactic treatment of ALDH2‐deficient mice before the start of ethanol consumption prevents bone loss induced by ethanol.^(^ ^26^ ^)^ To determine if AAVrh.10hALDH2 vector administration was able to treat established bone loss associated with ethanol consumption in ALDH2‐deficient mice, femurs from the mice were analyzed by μCT scans of trabecular bone and midshaft cortex.^(^ ^31^, ^32^, ^33^ ^)^ *Aldh2* ^E487K+/+^ mice evaluated after 6 weeks of ethanol consumption before receiving treatment had a significant reduction in cortical thickness compared with mice receiving only water (Fig. 3). After 18 weeks of ethanol consumption and 12 weeks post‐AAV vector administration, PBS and AAVrh.10control‐administered male and female mice continued to show significantly lower cortical thickness than *Aldh2* ^E487K+/+^ mice administered PBS but consuming water. However, both male and female mice treated with AAVrh.10hALDH2 showed a significant increase in cortical thickness compared with AAVrh.10control‐treated mice (males *p* < 0.05, females *p* < 0.001; Fig. 3*C, D*). A similar trend toward increased bone volume was observed for trabecular bone (Fig. 4*A–D*). Other trabecular parameters including trabecular number, thickness, and space were not significantly altered (Fig. 4*E–J*). No significant differences were found between any groups for mean density bone volume or cortical diameter (X or Y dimension; Supplemental Fig. S2).

**Fig. 5:**
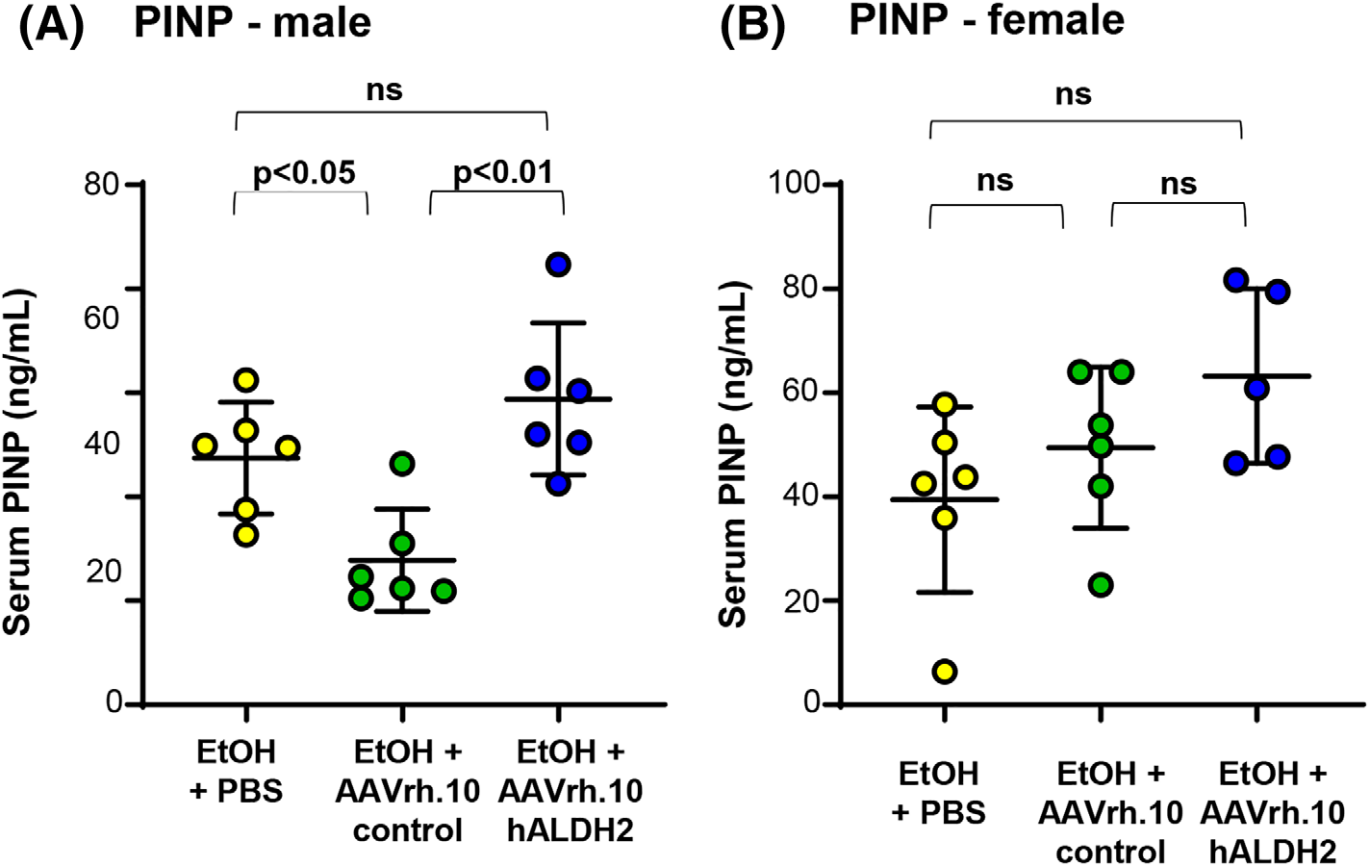
Serum N‐terminal propeptide of type I procollagen (PINP) levels. Mice were challenged with water or ethanol for 18 weeks. Six weeks after beginning ethanol administration, *Aldh2* ^E487K+/+^ mice were intravenously administered AAVrh.10hALDH2 (10^11^ gc), AAVrh.10control (10^11^ gc), or PBS. Serum levels of PINP were assessed at 6 weeks post‐vector administration (week 12 post‐initiation of ethanol challenge). (*A*) Males. (*B*) Females. Values are presented as means ± SD.

Bone turnover markers are proteins released by osteoclasts or osteoblasts during bone remodeling. We examined the level of bone formation marker N‐terminal propeptide of type 1 collagen (PINP) in serum taken at different time points. At 12 weeks, there was an increase in PINP levels in AAVrh.10hALDH2‐treated mice compared with mice administered AAVrh.10control (male *p* < 0.01, female not significant; Fig. 5). The observed increase in PINP is consistent with the increased cortical thickness observed by μCT.

## Discussion

ALDH2 deficiency is one of the most common hereditary disorders affecting ~8% of the world population and 35% to 45% of individuals of East Asian heritage.^(^ ^1^, ^2^, ^3^ ^)^ There are 67 million individuals worldwide with ALDH2 deficiency who are >50 years old and are at increased risk of osteoporosis that is further accelerated by chronic alcohol consumption.^(^ ^2^, ^3^, ^34^, ^35^, ^36^ ^)^ ALDH2 serves in the ethanol metabolism pathway to convert acetaldehyde to acetate predominantly in the liver. The common E487K mutation in ALDH2 reduces the oxidizing ability of the enzyme, resulting in increased levels of serum acetaldehyde.^(^ ^3^, ^8^, ^9^, ^10^, ^11^ ^)^ Acetaldehyde is a toxic intermediate causing the “Asian flush syndrome” and linked to increased risk of serious neurological, endocrine, cardiovascular, and dermatological disorders, altered drug metabolism, marked increase in risk for upper aerodigestive tract cancers, and osteoporosis.^(^ ^2^, ^3^, ^16^, ^17^, ^20^, ^23^, ^24^ ^)^ Previous studies from our lab demonstrated that prophylactic administration of ALDH2‐deficient mice with a liver‐directed gene therapy vector AAVrh.10hALDH2 could significantly prevent development of acute and chronic phenotypes related to ethanol consumption and ALDH2 deficiency, including osteopenia.^(^ ^26^, ^28^ ^)^ The current data demonstrate that after establishment of bone loss, therapeutic treatment with AAVrh.10hALDH2 replaced ALDH2 enzymatic activity in the liver and partially restored bone density in the femur even in the presence of further ethanol consumption.

### ALDH2, bone formation, and osteoporosis

The *ALDH2* ^E487K^ polymorphism (rs671; ALDH2*2 allele) is significantly associated with osteoporosis. The number of individuals diagnosed with osteoporosis is significantly higher for homozygous individuals compared with heterozygous or wild‐type individuals (odds ratio [OR] 3.33) after adjusting for other parameters, including alcohol consumption history.^(^ ^23^ ^)^ The ALDH2*2 allele is also significantly associated with hip fracture (OR 2.48) and osteoporosis (OR 2.04) when alcohol consumption was not considered.^(^ ^24^ ^)^

Studies in ALDH2‐deficient mice support a link between ALDH2, bone formation, and osteoporosis. Aldh2 knockout mice (*Aldh2* ^−/−^) given 5% ethanol for 4 weeks have significantly reduced trabecular bone volume and femoral bone mineral density compared with littermates given water or wild‐type mice.^(^ ^25^ ^)^ In the absence of ethanol, *Aldh2* ^−/−^ mice show no difference in trabecular bone mineral density but had increased cortical bone mineral density and thickness compared with wild‐type mice.^(^ ^37^ ^)^ Interestingly, climbing exercises that induce bone formation in wild‐type mice do not increase trabecular bone mineral density in *Aldh2* ^−/−^ mice because of impaired osteoblast differentiation due to altered bone formation signals in the knockout mice.^(^ ^38^, ^39^ ^)^ Transgenic mice expressing Aldh2*2 have reduced bone mineral density and bone mass alongside impaired osteoclast and osteoblast differentiation even in the absence of ethanol consumption, supporting a role for ALDH2 in bone homeostasis.^(^ ^20^ ^)^ In our previous study, *Aldh2* ^E48K+/+^ and *Aldh2* ^−/−^ mice given ethanol for 12 weeks showed significantly lower trabecular and cortical bone measurements in all parameters from μCT scans; however, in the absence of ethanol, there was no significant difference from wild‐type mice.^(^ ^26^ ^)^

### Role of ethanol and acetaldehyde in osteoporosis

Chronic alcohol use is associated with significant bone loss, osteoporosis, and an increased incidence of fracture.^(^ ^16^, ^18^, ^19^, ^40^ ^)^ Acetaldehyde, an intermediate of ethanol metabolism, interferes with bone metabolism. Ethanol and acetaldehyde both interfere with the formation of early osteoblast progenitors in mouse and human bone marrow cultures at physiologic concentrations found in alcoholics.^(^ ^17^ ^)^ Ethanol reduces human trabecular osteoblast proliferation and alkaline phosphatase activity in vitro.^(^ ^41^ ^)^ In a rat bone culture model, low amounts of acetaldehyde are a potent inhibitor of both bone formation and resorption.^(^ ^42^ ^)^ Similarly, Takeshima and colleagues^(^ ^24^ ^)^ found that osteoblast and osteoclast differentiation were significantly inhibited with acetaldehyde treatment of MC373E1 osteoblastic cells and mouse bone marrow‐derived osteoclast progenitors. The effects of acetaldehyde from chronic alcohol use on bone formation and homeostasis will be magnified in individuals with ALDH2 deficiency as the defective enzyme leads to increased systemic acetaldehyde compared with wild‐type individuals.^(^ ^3^, ^8^, ^9^ ^)^

### Modulation of ALDH2 and risk for osteoporosis

The role of ALDH2 in bone homeostasis and ethanol metabolism make it an attractive target for modulation to lower the risk of osteoporosis and fracture. In addition to acetaldehyde, ALDH2 metabolizes other endogenous aldehydes produced from lipid peroxidation and oxidative stress such as 4‐hydroxynonenal (4‐HNE).^(^ ^2^, ^43^ ^)^ Enhanced oxidative stress is associated with bone loss through an imbalance in the bone remodeling process.^(^ ^44^ ^)^ In vitro, the inhibition of osteoblast differentiation and proliferation by the ALDH2*2 allele or acetaldehyde application is reversed by treatment with the antioxidant vitamin E analog Trolox C.^(^ ^20^ ^)^ Another antioxidant, astaxanthin, improves impaired osteoblast differentiation in Aldh2*2 transgenic or acetaldehyde‐treated osteoblasts and increased femur bone density in Aldh2*2 transgenic mice.^(^ ^45^ ^)^ Alda‐1, a small molecule activator of ALDH2, enhances ALDH2 activity in osteoblasts in vitro and in vivo and promotes osteoblast differentiation by increasing bone morphogenic protein‐2 expression.^(^ ^46^, ^47^ ^)^ In vivo rat studies with Alda‐1 demonstrated enhanced bone regeneration at the site of fracture and the reversal of trabecular bone loss in ovarectomized osteoporotic rats.^(^ ^46^, ^47^ ^)^ In contrast, disulfram (Antabuse), an ALDH2 inhibitor used to treat alcohol use disorders, causes trabecular osteopenia in adult rats, diminished bone regeneration at fracture sites, and stunted bone formation as measured by histomorphometry.^(^ ^47^, ^48^ ^)^

Prophylactic gene therapy using AAVrh.10hALDH2 restored wild‐type ALDH2 function in the liver and reduced systemic acetaldehyde and liver malondialdehyde levels after acute and chronic alcohol use in ALDH2‐deficient mice.^(^ ^26^, ^28^ ^)^ When administered before beginning ethanol consumption, AAVrh.10hALDH2 therapy prevents bone loss in the ALDH2‐deficient mice.^(^ ^26^ ^)^ In the present study, we have extended these observations to demonstrate that AAVrh.10hALDH2 treatment after osteopenia establishment significantly restores femur cortical bone in ALDH2‐deficient male and female mice drinking chronic ethanol. Thus, reversal of bone loss was achieved even in the face of continued insult from chronic ethanol. Cortical bone is the most important type of bone in the resistance to fractures.^(^ ^49^, ^50^, ^51^ The magnitude of increase in cortical bone thickness was greater than achieved with FDA‐approved parathyroid hormone (PTH) treatment in a mouse model of postmenopausal osteoporosis.^(^ ^52^ ^)^ As a large number of individuals carrying an inactive ALDH2*2 allele are also regular or heavy drinkers,^(^ ^53^, ^54^ ^)^ there is a large population at increased risk of developing osteoporosis.^(^ ^2^, ^3^, ^17^, ^20^ ^)^ AAVrh.10hALDH2 administration after osteopenia was  established increased femur cortical bone thickness  and showed a trend toward increased trabecular bone volume. There remains a particular continued need for therapies that increase long bone cortical bone mass given that existing osteoporosis therapies, such as PTH analogues, typically have a more robust effect on vertebral bone over long bones.^(^ ^55^ ^)^ Thus, ALDH2 gene therapy could be used to mitigate the detrimental effects on quality of life and the financial burden as both a prophylactic and therapeutic treatment for bone loss caused by chronic alcohol use with ALDH2 deficiency. Because of the role of ALDH2 in normal bone homeostasis, ALDH2 gene therapy with AAVrh.10hALDH2 may also benefit ALDH2‐deficient individuals with osteoporosis who are not chronic alcohol users.

## Conflicts of Interest

RGC has equity in and is a consultant to LEXEO Therapeutics, and RGC and OEP are participants in a patent disclosure regarding gene therapy for ALDH2 deficiency. All other authors state that they have no conflicts of interest.

### Peer Review

The peer review history for this article is available at https://publons.com/publon/10.1002/jbm4.10723.

## Supporting information


**Data S1.** Supporting Information.
