# Supplementary material for: Gene Therapy to Treat Osteopenia Associated With Chronic Ethanol Consumption and Aldehyde Dehydrogenase 2 Deficiency
Source: JBMR Plus. 2023 Feb 16;7(4):e10723. doi: 10.1002/jbm4.10723 (PMC10097638; doi:10.1002/jbm4.10723)
Supplement: Supplementary file 1 — Data S1. Supporting Information. [file JBM4-7-e10723-s001.pdf]

## Supplemental Table I. Statistical Values of Change in Body Weight from Baseline at Each Time Point Comparing All Groups of Male Mice[1]

| **Week** | **PBS + water *vs* PBS + EtOH** | **PBS + water *vs* AAVrh.10control + EtOH** | **PBS + water *vs* AAVrh.10hALDH2 + EtOH** | **PBS + EtOH *vs* AAVrh.10control + EtOH** | **PBS + EtOH *vs* AAVrh.10hALDH2 + EtOH** | **AAVrh.10control + EtOH *vs* AAVrh.10hALDH2 + EtOH** |
|---|---|---|---|---|---|---|
| 0 | ns | ns | ns | ns | ns | ns |
| 1 | -- | -- | -- | ns | ns | ns |
| 2 | -- | -- | -- | ns | ns | ns |
| 3 | -- | -- | -- | ns | ns | ns |
| 4 | -- | -- | -- | ns | ns | ns |
| 5 | <0.002 | <0.0001 | <0.0001 | ns | ns | ns |
| 6 | -- | -- | -- | ns | ns | ns |
| 7 | -- | -- | -- | ns | ns | ns |
| 8 | -- | -- | -- | ns | ns | ns |
| 9 | <0.0001 | <0.0001 | <0.0001 | ns | ns | ns |
| 10 | -- | -- | -- | ns | ns | ns |
| 11 | -- | -- | -- | ns | ns | ns |
| 12 | <0.0001 | <0.0001 | <0.0001 | ns | <0.05 | ns |
| 13 | -- | -- | -- | ns | <0.01 | <0.05 |
| 14 | -- | -- | -- | ns | <0.001 | <0.05 |
| 15 | <0.0001 | <0.0001 | <0.0001 | ns | <0.0001 | <0.01 |
| 16 | -- | -- | -- | ns | <0.0001 | <0.0001 |
| 17 | -- | -- | -- | ns | <0.0001 | <0.0001 |
| 18 | <0.0001 | <0.0001 | <0.0001 | ns | <0.0001 | <0.0001 |

[1] A two-way ANOVA with Tukey's multiple comparison test was used to compare means of each group at each time point.

[2] ns – not significant

**Supplemental Table II. Statistical Values of Change in Body Weight from Baseline at Each Time Point Comparing All Groups of Female Mice[1]**

| Week | PBS + water *vs* PBS + EtOH | PBS + water *vs* AAVrh.10control + EtOH | PBS + water *vs* AAVrh.10hALDH2 + EtOH | PBS + EtOH *vs* AAVrh.10control + EtOH | PBS + EtOH *vs* AAVrh.10hALDH2 + EtOH | AAVrh.10control + EtOH *vs* AAVrh.10hALDH2 + EtOH |
|---|---|---|---|---|---|---|
| 0 | ns | ns | ns | ns | ns | ns |
| 1 | -- | -- | -- | ns | ns | ns |
| 2 | -- | -- | -- | ns | ns | ns |
| 3 | -- | -- | -- | ns | ns | ns |
| 4 | -- | -- | -- | ns | ns | ns |
| 5 | <0.0001 | <0.0001 | <0.0001 | ns | ns | ns |
| 6 | -- | -- | -- | ns | ns | ns |
| 7 | -- | -- | -- | ns | ns | ns |
| 8 | -- | -- | -- | <0.05 | ns | ns |
| 9 | <0.0001 | <0.0001 | <0.0001 | ns | ns | ns |
| 10 | -- | -- | -- | ns | ns | ns |
| 11 | -- | -- | -- | ns | ns | ns |
| 12 | <0.0001 | <0.0001 | <0.0001 | ns | ns | ns |
| 13 | -- | -- | -- | ns | <0.05 | ns |
| 14 | -- | -- | -- | ns | <0.05 | ns |
| 15 | <0.0001 | <0.0001 | <0.0001 | <0.05 | <0.0001 | ns |
| 16 | -- | -- | -- | ns | <0.0001 | <0.0001 |
| 17 | -- | -- | -- | ns | <0.0001 | <0.0001 |
| 18 | <0.0001 | <0.0001 | <0.0001 | ns | <0.0001 | <0.0001 |

[1] A two-way ANOVA with Tukey's multiple comparison test was used to compare means of each group at each time point.

[2] ns – not significant

**Supplemental Table III. Statistical Values of Locomotion Test at Each Time Point Comparing All Groups of Male Mice[1]**

| Week | PBS + water *vs* PBS + EtOH | PBS + water *vs* AAVrh.10control + EtOH | PBS + water *vs* AAVrh.10hALDH2 + EtOH | PBS + EtOH *vs* AAVrh.10control + EtOH | PBS + EtOH *vs* AAVrh.10hALDH2 + EtOH | AAVrh.10control + EtOH *vs* AAVrh.10hALDH2 + EtOH |
|---|---|---|---|---|---|---|
| 0 | ns | ns | ns | ns | ns | ns |
| 5 | ns | ns | <0.05 | ns | ns | ns |
| 9 | ns | ns | ns | ns | <0.05 | <0.05 |
| 12 | <0.01 | <0.05 | ns | ns | <0.05 | ns |
| 15 | <0.0001 | <0.001 | ns | ns | <0.01 | ns |
| 18 | <0.0001 | <0.01 | ns | ns | <0.01 | ns |

[1] A two-way ANOVA with Tukey's multiple comparison test was used to compare means of each group at each time point.

[2] ns – not significant

**Supplemental Table IV. Statistical Values of Locomotion Test at Each Time Point Comparing All Groups of Female Mice[1]**

| Week | PBS + water *vs* PBS + EtOH | PBS + water *vs* AAVrh.10control + EtOH | PBS + water *vs* AAVrh.10hALDH2 + EtOH | PBS + EtOH *vs* AAVrh.10control + EtOH | PBS + EtOH *vs* AAVrh.10hALDH2 + EtOH | AAVrh.10control + EtOH *vs* AAVrh.10hALDH2 + EtOH |
|---|---|---|---|---|---|---|
| 0 | ns | ns | ns | ns | ns | Ns |
| 5 | <0.05 | ns | <0.05 | ns | ns | ns |
| 9 | ns | ns | ns | ns | ns | ns |
| 12 | <0.01 | <0.01 | ns | ns | ns | ns |
| 15 | <0.05 | <0.05 | ns | ns | ns | ns |
| 18 | ns | <0.05 | ns | ns | ns | ns |

[1] A two-way ANOVA with Tukey's multiple comparison test was used to compare means of each group at each time point.

[2] ns – not significant

## Supplemental Figure Legends

**Supplemental Figure 1.** Effect of AAVrh.10hALDH2 therapy on body weight during chronic ethanol exposure. Mice were challenged with water or ethanol for 18 wk. Six wk after initiation of ethanol administration, *Aldh2*$^{E487K+/+}$ mice were intravenously administered AAVrh.10hALDH2 ($10^{11}$ gc), AAVrh.10control ($10^{11}$ gc), or PBS. Mice were weighed every week. **A.** Body weight - males; **B**. Body weight – females.

**Supplemental Figure 2.** Three dimensional μCT reconstructions of femoral cortical bone for AAVrh.10hALDH2 therapy after chronic ethanol ingestion. Aldh2$^{E487K+/+}$ mice were challenged with water or ethanol for 18 wk in total. Six wk post-initiation of ethanol administration, mice were intravenously administered AAVrh.10hALDH2 ($10^{11}$ gc), AAVrh.10control ($10^{11}$ gc), or PBS. Fixed femurs were analyzed by μCT. **A.** Mean density bone volume – males; **B.** Mean density bone volume – females. Cortical diameter measured at same site as cortical thickness (Cb.Th) measurement. **C.** Cortical diameter (X dimension) – males; **D.** Cortical diameter (X dimension) – females; **E.** Cortical diameter (Y dimension) – males; **F.** Cortical diameter (Y dimension) – females.

Supplemental Figure 1

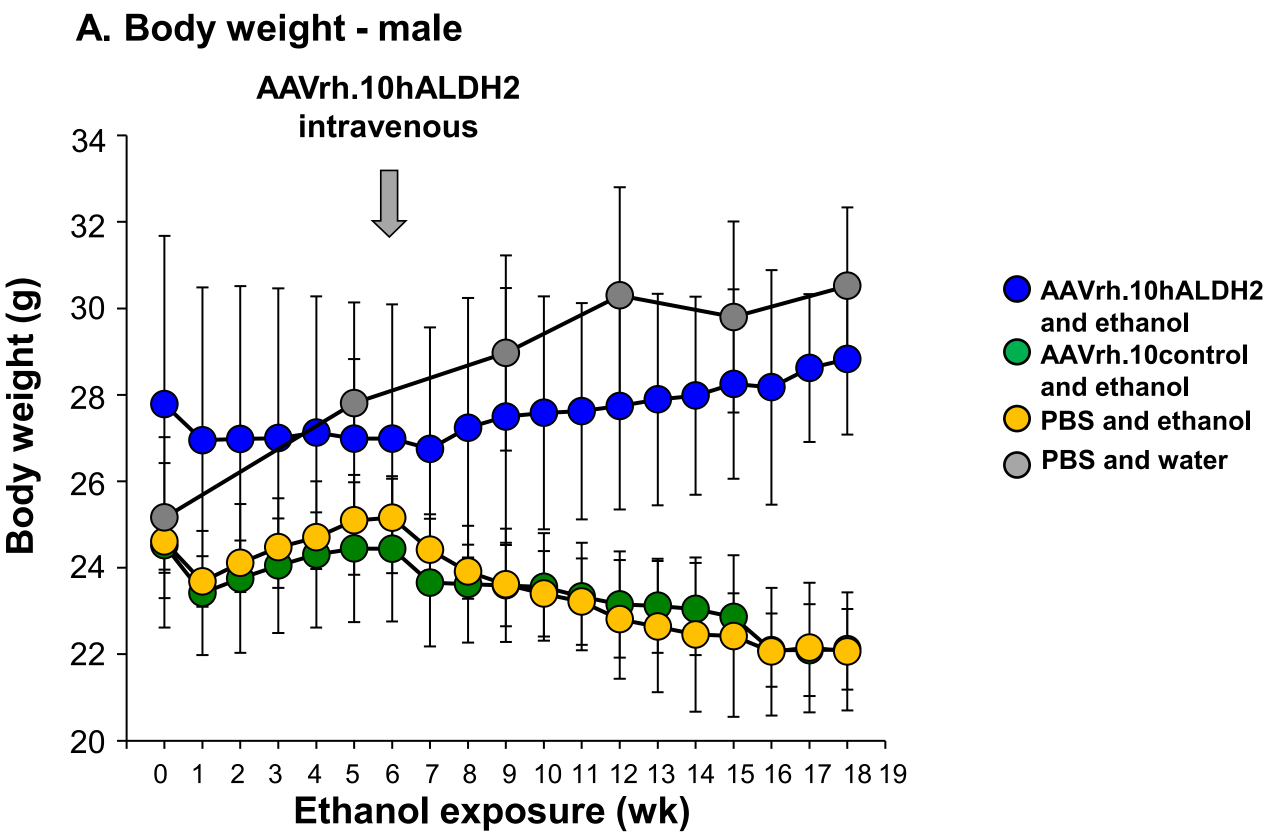


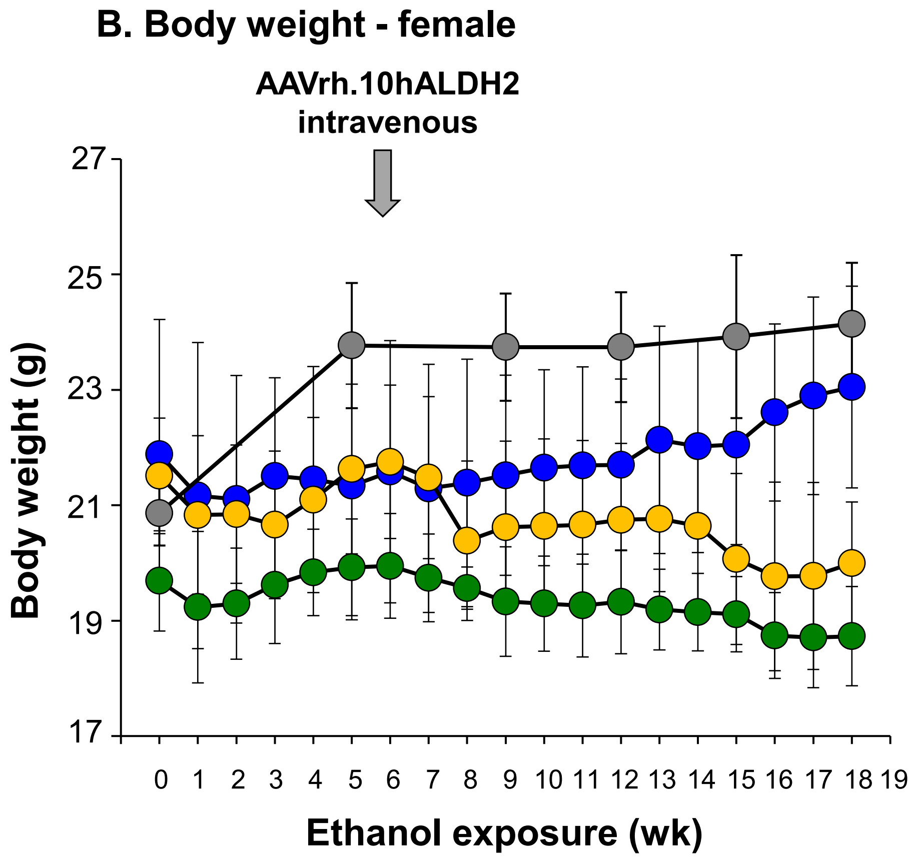

Supplemental Figure 2A, B

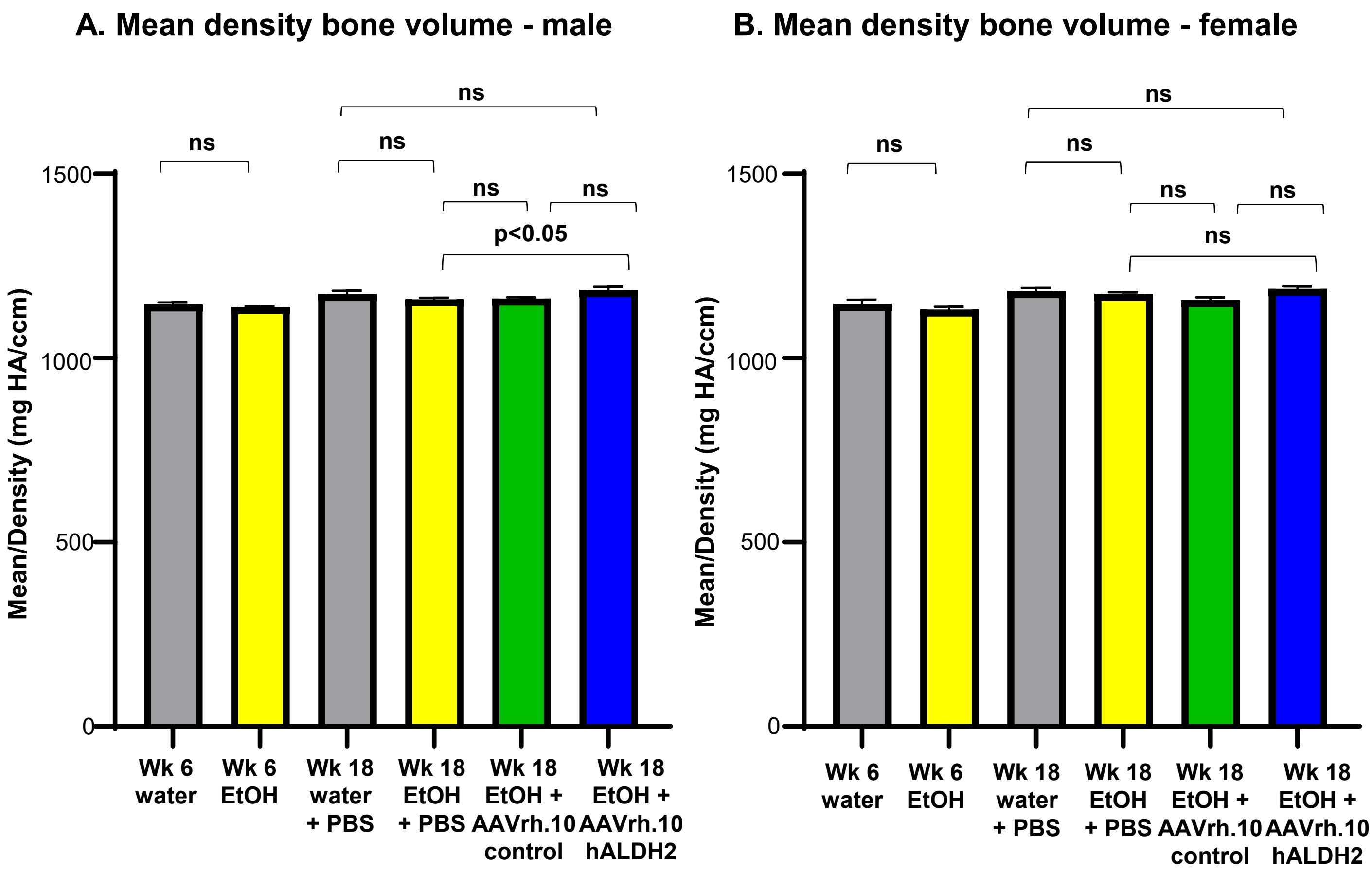

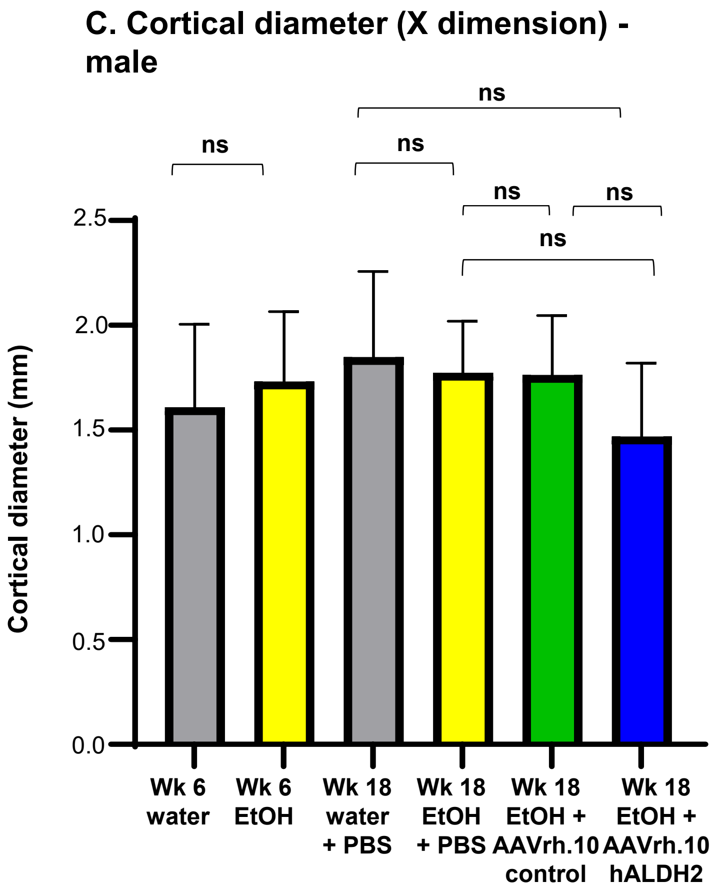
C. Cortical diameter (X dimension) - male
ns
ns
ns
ns
ns
ns
Cortical diameter (mm)
2.5
2.0
1.5
1.0
0.5
0.0
Wk 6 water
Wk 6 EtOH
Wk 18 water + PBS
Wk 18 EtOH + PBS
Wk 18 EtOH + AAVrh.10 control
Wk 18 EtOH + AAVrh.10 hALDH2

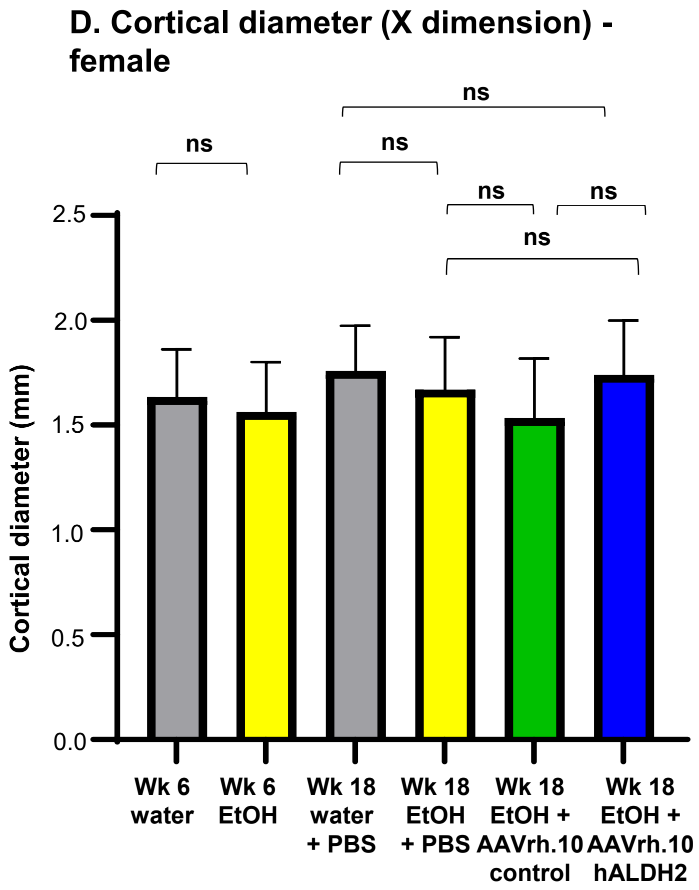
D. Cortical diameter (X dimension) - female
ns
ns
ns
ns
ns
ns
Cortical diameter (mm)
2.5
2.0
1.5
1.0
0.5
0.0
Wk 6 water
Wk 6 EtOH
Wk 18 water + PBS
Wk 18 EtOH + PBS
Wk 18 EtOH + AAVrh.10 control
Wk 18 EtOH + AAVrh.10 hALDH2

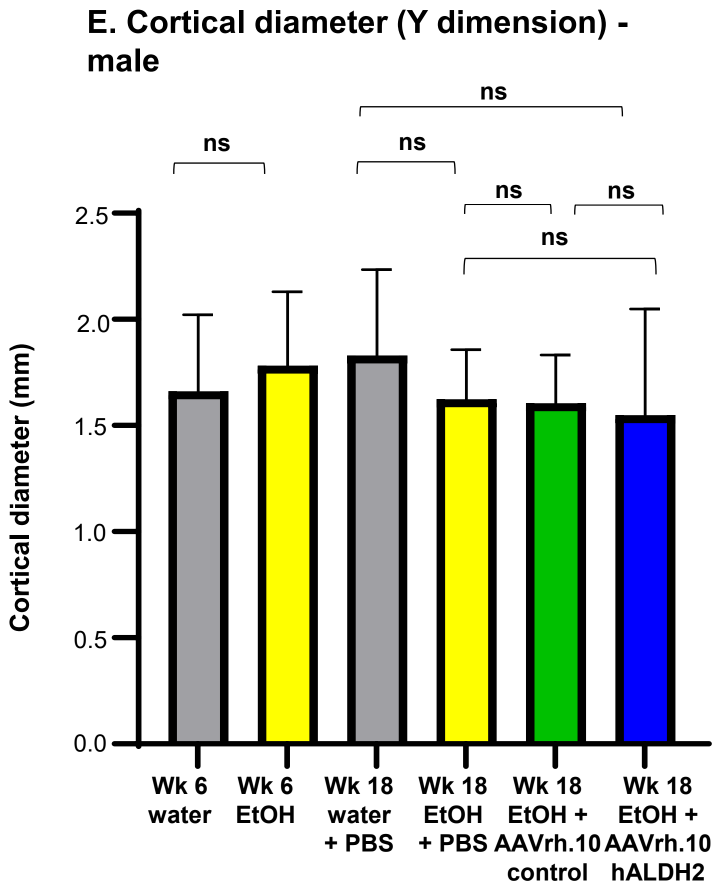
E. Cortical diameter (Y dimension) - male
ns
ns
ns
ns
ns
ns
Cortical diameter (mm)
2.5
2.0
1.5
1.0
0.5
0.0
Wk 6 water
Wk 6 EtOH
Wk 18 water + PBS
Wk 18 EtOH + PBS
Wk 18 EtOH + AAVrh.10 control
Wk 18 EtOH + AAVrh.10 hALDH2

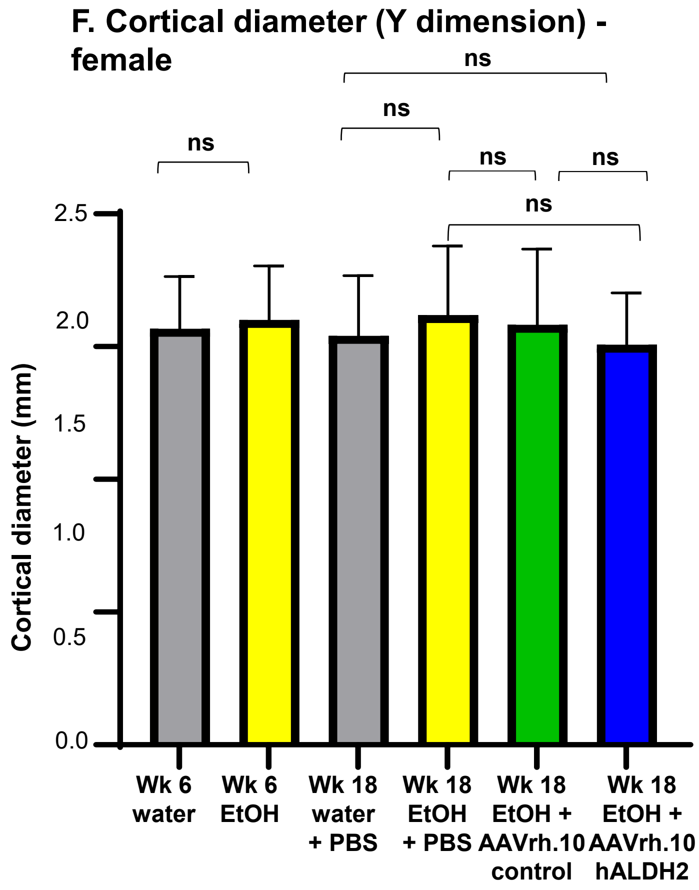
F. Cortical diameter (Y dimension) - female
ns
ns
ns
ns
ns
ns
Cortical diameter (mm)
2.5
2.0
1.5
1.0
0.5
0.0
Wk 6 water
Wk 6 EtOH
Wk 18 water + PBS
Wk 18 EtOH + PBS
Wk 18 EtOH + AAVrh.10 control
Wk 18 EtOH + AAVrh.10 hALDH2
